# Challenges and recommendations for conducting epidemiological studies in the field of epilepsy pharmacogenetics

**DOI:** 10.4103/0971-6866.80351

**Published:** 2011-05

**Authors:** Sandeep Grover, Meenal Gupta, Ritushree Kukreti

**Affiliations:** Institute of Genomics and Integrative Biology (IGIB), Council of Scientific and Industrial Research (CSIR), Mall Road, Delhi, India

**Keywords:** Epilepsy, pharmacogenomics, phenotype, sample size, study design

## Abstract

Epilepsy is one of the most prevalent neurological disorders, afflicting approximately 50 million Indians. Owing to affordability and easy availability, use of first-generation antiepileptic drugs (AEDs) is heavily encouraged for the treatment of epilepsy in resource-limited countries such as India. Although first-generation AEDs are at par with second-generation AEDs in terms of efficacy, adverse drug reactions (ADRs) are quite common with them. This could be attributed to the inferior pharmacokinetic parameters such as nonlinear metabolism, narrow therapeutic index and formation of toxic intermediates. In addition, epilepsy patients may differ in the pharmacokinetic and pharmacodynamic profiles, with about 1/3^rd^ of the population failing to respond to treatment. A proportion of this interindividual variability in response may be explained by genetic heterogeneity in the activity and expression of the network of proteins such as metabolizing enzymes, transporters and targets of AEDs. Over the last two decades, a considerable effort has been made by the scientific community for unraveling this genetic basis of variable response to AEDs. However, there have been inconsistencies in such genetic association studies conducted across different territories of the world. There could be several reasons underlying the poor replicability of these studies, mainly nonuniform phenotypic definitions, poor sample size and interethnic variability. In the present review article, we provide an overview of heterogeneity in study designs for conducting pharmacogenetic studies. In addition, critical recommendations required for overcoming such challenges imposed by pharmacogenetic epidemiological studies have been briefly discussed.

## Introduction

Epilepsy, characterized by recurrent unprovoked seizures, is one of the most common brain disorders. However, commonly available AEDs are effective in only 60–70% of the epilepsy patients and are often associated with ADRs. Pharmacogenetic studies may provide vital clues for providing optimal beneficial treatment with minimum risk for developing drug-related side-effects. Post the Human Genome Project, with the advent of high-throughput genotyping chips, genetic studies have garnered enormous attention and are increasingly being used to identify genetic variants that might influence drug response and predisposition to ADRs in patients on AEDs.[1] Prominent among these sequence variants are millions of single nucleotide polymorphisms (SNPs), which have emerged as strong candidates for drug-response studies. The availability of such an enormous wealth of data has served to fuel the pharmacogenetic epidemiological studies. However, such studies often come under the scanner owing to a lack of reproducibility of the results. There are several key issues in this regard which need to be adequately addressed to ensure the validity, accuracy and reliability of such results before coming to scientifically relevant conclusions. These issues could range from population stratification, smaller sample size, inconsistency in phenotypic definition across different studies, highly heterogeneous clinical symptoms in a specific study design and unaccountability of all the environmental variables.[2] Further, selection and prioritization of candidate genes and SNPs and use of appropriate statistical tools could also play a major factor in detecting true positive associations. We provide an assessment of variability in study designs, accountability of confounding factors and use of bioinformatics and statistical tools, with an emphasis on the pharmacogenetic studies of epilepsy. Looking at such differences in methodological issues may help us to resolve inconsistencies in replication studies and in extending the laboratory findings to clinical practice.

## Clinical study design and phenotypic data collection

A prerequisite for a successful genetic study is a large cohort of patient samples with well-defined phenotypes, especially in case of complex genetic disorders including epilepsy. One of the pressing challenges faced by geneticists today is the clinical heterogeneity of seizure and syndrome types as well as the associated etiology.[3] In addition, complexity in the classification and terminology of epilepsies makes it highly unlikely that it will be used for non-diagnostic purposes.

To confront this serious issue, several research groups have evolved their own phenotypic classifications based on parameters such as seizure frequency, time to first seizure, time to seizure remission, time to drug withdrawal and number of drugs tried.[4–6] Further, time period for evaluation of these parameters vary considerably from 3 to 12 months.[4–6] In addition, there are several other key issues that need to be addressed for uniformity in study designs. Most pharmacogenetic studies have failed to exclude patients with symptomatic epilepsies that may confound clinical outcome by rendering patients to respond poorly to AED treatment, irrespective of type, dose and duration of drug therapy. In addition to nature and regimen of drug therapy, brand of the drug administered, concurrent hormonal therapy and history of treatment could all have a major impact on the improvement in clinical symptoms or phenotypes under observation during the course of the study.[7] Furthermore, stratification according to gender and age are crucial for conducting epidemiological studies.[7] The most common phenotype for the drug response studies have been drug resistance to AEDs. However, such studies may not yield meaningful interpretations owing to the trial of multiple drugs on the same patients, as AED–AED interaction is a fairly common observation in epilepsy patients.[7] Very limited studies have attempted to garner pharmacogenetic data on monotherapy epilepsy patients.[4] Hence, there is a need to develop the concept of endophenotypes with the purpose of dividing disease symptoms into more stable phenotypes, eventually leading to a robust study design with more powerful test statistics.[8] In summary, all the variables that directly or indirectly play a role in influencing phenotypic characteristics of a patient must be given appropriate weightage before deciding upon the inclusion and exclusion criteria for enrolling a patient. Further, measurement of these clinical parameters must be included in the study design, with accountability in statistical analysis and interpretation.

## Prioritizaion of genes and genetic loci

Recent genetic association studies have adapted both the candidate gene approaches as well as the genome-wide association (GWA) studies for identification of associated genetic variants. There are several popular strategies for candidate gene selection, including positional and functional approaches. The former relies on the linkage-based approach, which does not require any assumptions regarding the disease mechanism. Chromosomal regions found in high linkage with the drug response are exploited to search for relevant genes residing at that locus using LD gene mapping. A functional approach, on the other hand, requires a preformed hypothesis indicating a direct or indirect role of the candidate gene in a pathway/physiological process relevant to the disease phenotype and disposition of drugs. For instance, genes encoding enzymes involved in the metabolism of AEDs, such as *CYP2C9, CYP2C19, EPHX1* and *UGT2B7*, serve as prime candidate genes for testing the influence of genetic variability on variable drug response.[1,9,10] So far, most of the studies have focused on the role of functional alleles from these genes on decrease or increase in metabolism of AEDs. However, studies exploring direct role of these variants on seizure control are very limited, with most of them focusing on transporter genes (*ABCB1, ABCC1* and *ABCC2*) and drug targets such as sodium channels (*SCN1A*).[1,9,10] This could be due to the difficulty in measuring the levels of AEDs to which different brain regions are exposed. In addition, the task of differentiating between sensitivity of drug targets and permeability of blood–brain barrier in influencing drug response is difficult and complicated. Another approach involves a study of genome-wide single nucleotide polymorphisms (SNPs) in a case–control study design for generating unbiased information. However, GWA studies have their own limitations, including a lack of cost-effectiveness, multiple hypothesis testing and the large sample size required for robust high-powered studies complemented with the fact that the available output from GWA studies explains only a fraction of disease heritability.[11] Recently, a meta-analysis of several GWA studies has gained considerable significance for the identification of disease-susceptible loci with a higher confidence. So far, GWA studies evaluating drug response in epilepsy patients are lacking, owing to poor sample size in different phenotypic categories with a minimum requisite of hundreds, if not thousands, of epilepsy patients in each group. In recent times, studies are now taking advantage of large-scale deep resequencing to develop a better understanding of the human genome, and it is very likely that such approaches will be used in the future for pharmacogenomic studies.

## Use of public SNP resources and bioinformatics tools

With the swiftly evolving databases and state-of-the-art tools, bioinformatics is rapidly becoming an integral part of the study design, wherein concerted efforts of computational biologists and geneticists might help in converting the sequence information into a better understanding of the biological processes. The completions of the Human Genome and the HAPMAP projects have created abundant data in the form of complete human genome sequence and the genetic variations. However, this information, dispersed across different locations, is futile until integrated and analyzed, thus compelling the development and expansion of essential bioinformatics resources and value-added databases to bridge the gap between the information available and the knowledge. Current bioinformatics resources, which provide a platform for data storage, retrieval and sharing along with analytical methods and algorithms, have become indispensable in genetic studies for the search of critical candidate genes and genetic variants. The major genome browsers, including the National Centre for Biotechnology Information (NCBI), European Molecular Biology Lab (EMBL) and University of Southern California Santa Cruz (UCSC), provide nucleotide sequences wherein most of the genes have been annotated and gene maps are available.[12] These are nonstatic databases that also provide the option for integration of data from subsequent projects and individual experiments. The gene prediction programs are continually updated to scan the sequence for general properties of protein-coding sequences, while others search all available sequence databases for homology to known genes from other organisms. The analysis may also allow the function of a new human gene to be deduced if its structure is homologous to a gene of known function in another organism. The databases also have information on the expression pattern of genes in tissues and organs, which may provide further clues to possible function. Further, “interactome databases,” such as KEGG PATHWAY and Reactome databases, provide a knowledgebase for different biological pathways. Availability of such wealth of data has significantly eased the selection of critical genes for disease susceptibility and drug-response studies. Further, databanks such as Online Mendelian Inheritance in Man (OMIM) and Orphanet provide a comprehensive summary of the information available for Mendelian disorders and correlated genes from previous studies. The SNP database (dbSNP) module of NCBI provides a platform that incorporates the information of SNPs, microsatellites, insertions and deletions from several sources including the HAPMAP project. Other SNP databases include Ensembl, Human Mutation Database (HMD), Japanese SNP database (JSNP), etc.

The advent of additional modules and algorithms that predict the function of the SNPs is increasingly being appreciated as they lead to increased probability of hitting the causal/functional variant rather than an associated or linked variant. For example, Polydoms site uses information from both dbSNP and “Gene SNPs” to provide a graphic display of gene synonymous and nonsynonymous variations. F-SNP database provides integrated information about the functional effects of SNPs predicted at the transcriptional, splicing, translational and post-translational levels obtained from 16 different bioinformatics tools and databases.[12] The other tools available include Fast-SNP, SIFT, LS-SNP, SNPeffect, SNPs3D, PolyPhen, ESEfinder, ESRSearch, PESX, TFSEARCH, Consite, GoldenPath, OGPET, Sulfinator and KinasePhos, some of which are also used by F-SNP. Furthermore, the HAPMAP project provides frequencies of over four million SNPs in four different populations from Africa, China, Utah and Japan. Bioinformatics tools such as Tagger can use the Linkage Disequilibrium (LD) information provided by the HAPMAP project to identify SNPs in high LD. The SNPs in tight linkage yield the same information and usage of representative SNP per LD block (Tag SNPs), reduces the cost and information load.[12] However, LD transferability between different populations might be a major limitation. On the off-side, a major challenge faced by bioinformaticians is the lack of consistent information between the different databases. For instance, the differences in annotation methods used by different genome browsers lead to discrepancy in the gene information available. More frequently, the number of SNPs displayed per gene differs between different databases. The collaborative consensus coding sequence (CCDS) project reflects the first step taken in this direction. The project was undertaken with the aim of identifying a common protein-coding gene set for the human and mouse genomes.[13] The efforts undertaken have led to consistent representation of gene information across NCBI, Ensembl and UCSC genome browsers, which is essential to maintain a high standard of reliability and biological accuracy. Further, online databases such as HuGE Navigator or the NIH Genetic Association Database and The Epilepsy Genetic Association Database (epiGAD) provide the options for systematic data tabulation and display, highly relevant for epilepsy researchers with detailed information such as protective and risk-alleles, epilepsy syndrome, study duration and sample size.[14]

### 

#### Statistical analysis and interpretation

Statistical analysis plays a fundamental role in interpreting the findings of complex genetic research, and several statistical issues need to be addressed during the study design stage itself to prevent erroneous results. In this section, we discuss some key statistical issues of interest in genetic study designs.

## Hardy-Weinberg equilibrium

The Hardy-Weinberg equilibrium (HWE) equation allows prediction of genotype frequencies in a population if the allele frequencies are known, based on the assumption that large, randomly mating populations do not show changes in genotype and allele frequencies over one generation after another in the absence of natural selection, mutations, genetic drift immigration and emigration. It is thus an important tool to scrutinize selection in ethnically diversified populations. In a case–control setup, the control genotypes are expected to be in conformance with HWE. The most common causes of deviations from HWE are genotyping error or population stratification.[15] Deviation from HWE in cases in the absence of these confounding effects can provide evidence for association, wherein the true genetic effect of the SNP is not controlled by a multiplicative model.[15] However, because the affected samples are over-represented in such studies than that are expected in a random population, there is a good probability that inflated type-I error in HWE tests might result in exclusion of potential markers from the study.[15]

### 

#### Population stratification

Population stratification has become a crucial statistical issue as it can lead to spurious results, especially in a case–control study design. Stratification refers to the existence of subpopulations with different allele frequencies that might be a result of founder effects, genetic drift or recent admixture.[16] Association studies with such unmatched subjects might result in statistical associations between a disease phenotype and arbitrary markers that have no physical linkage to the causative loci. Exclusion of stratification is therefore more of a necessity than an option in a case–control association study and requires the recruitment of subjects from a genetically homogeneous population. In addition, several tools and algorithms have been devised in order to check the population stratification in such studies. Pritchard and Rosenberg *et al*. proposed the use of a set of unlinked markers that are unrelated to the disease or the drug response.[16] These unlinked markers will not exhibit significant differences in genotype/allele frequencies between the responder and the nonresponder groups of the study (that would be expected in case of population stratification) as well as in the control individuals. Genomic control (GC) approaches had been proposed to adjust for the confounding effects of population stratification, but are less sensitive for moderate stratification and subtle substructures within the studied population.[17] In addition, statistical tools such as structure, principal component analysis (PCA) and multidimensional scaling (MDS) have proved effective and are commonly used to address this issue.[17]

## Power and sample size

Another vital limb of a genetic study is computation of statistical power. Power of association studies refers to the probability of correctly detecting a genuine association, and is often estimated before carrying out the study to determine the sample size required for finding a true genetic effect.[18] Most studies aim to achieve a power of 80%, and the predicted sample sizes required to do so depend largely on the effect size of the genetic variants under evaluation. Other factors that influence the power of a study include local patterns of LD, allele frequency differences between marker and trait loci, allelic and genetic heterogeneity, required level of statistical significance (α) and genotyping errors.

Although sample sizes for conducting pharmacogenetic studies in epilepsy have increased in recent years, majority of the studies had a sample size of 200 or less epilepsy patients for both the case and the control groups.[14] Such a diminished sample size may result in weak positive association studies which are often difficult to replicate.

### 

#### Linkage Disequilibrium

LD, which describes the correlation/association between genetic markers, is an important indicator of ancestral recombination. This non-random association of markers exists over long distances across the chromosome interrupted by LD breaks. Several factors contributing toward LD include mutations, genetic drift, population admixture and ethnic diversity. On the other hand, recombination, gene conversion and recurrent mutations result in a reduction of LD. As a result, the LD patterns can vary significantly between populations.[19] Association studies do not always result in identification of causal/functional variant; often, markers in strong LD with causal variants are detected, which can then be used to identify the latter. Nevertheless, functional characterization of associated SNPs is an important step to validate the causal effects of the SNPs. With the availability of dense SNP maps, LD information presents an opportunity of selecting fewer representative SNPs (Tag SNPs) based on LD blocks that extend over considerable distances. This offers an advantage of selecting fewer SNPs, reducing the cost of genotyping and the information load. Usage of Tag SNPs might be more convenient once genome diversity and tag transferability across populations has been determined.

#### Beyond single SNP associations: Haplotype study, gene–gene and gene–environment interactions

The likelihood that concurrent effect of more than one SNP might influence the protein/enzyme activity necessitates extensive study of the relationship between the genetic loci, especially because reports have shown that the SNPs might not just have additive or synergistic effects but can also exhibit compensatory mechanisms to rescue or decrease the protein/enzyme activity. In addition, there are a large number of genes that could theoretically contribute to the interindividual variability in drug response in epilepsy, and such studies can be effectively carried out only if all such genes are considered together. Even among CYPs, genetic variants from *CYP1A2, CYP3A4, CYP2C8, CYP2C9* and *CYP2C19* could potentially contribute collectively to an altered drug metabolism phenotype in both monotherapy as well as multitherapy epilepsy patients hence emphasizing the need for study of multiple variants in the form of haplotype and gene–gene interaction studies that seems important. Studies have shown that while, often, single SNPs show moderate effects, haplotype-based studies have proven to be more powerful in detecting associations.[20] Population-based strategies rely on reconstruction of haplotypes from unphased genotype data of unrelated individuals. Several statistical software packages based on parsimony, maximum likelihood or Bayesian approaches have been devised for haplotype reconstruction. Furthermore, growing evidence indicates that susceptibility to diseases is influenced by underlying genetic pathway architecture, and the study of epistatic influences in pathway genes presents the potential to identify complex biological relationships. It can be envisaged that variants existing in genes lying upstream and/or downstream of a particular candidate gene, which lead to putative functional alterations, might magnify its influence several fold. Popular approaches undertaken by current studies include regression and dimensionality reduction methods.[21] An advantage of epistatic studies over single SNP association studies is that variants with marginal effects can be uncovered, which might exert their effect by interacting with other polymorphisms. The role of environmental factors in drug response cannot be ignored. Environmental factors might influence gene expression and may lead to augmenting or masking of the subtle differences caused by genetic variations. Such effects might be responsible for the varied results obtained from individual studies incorporating geographically and ethnically diverse populations. Such gene–environment interactions are mostly difficult to model, and major limitations include the requirement of significantly large samples with carefully defined exposures and similar clinical phenotypes, inability to unravel additive effects and failure of statistical interactions to reflect biological interactions.

## Multiple corrections

Another key issue in association studies is determination of threshold for significant results. Although nominal significance levels of 5% is generally acceptable, it might lead to inflated type I error, i.e. detection of false-positives when multiple independent tests are performed. One of the earliest tests proposed to overcome this limitation was Bonferroni correction, wherein the probabilities were recalculated depending on the number of independent tests performed.[22] However, the Bonferroni correction method has received much criticism as it is overly conservative. The studied SNPs in an association study might not be entirely independent; rather, they could be correlated and existing in LD. This might result in inflated type II error, i.e. increase in false-negatives and hence loss of results. Another method, Nyholt’s method for multiple corrections, takes into account the background LD for calculation of significance thresholds, but is still conservative in conditions of moderate LD.[23] Other popular methods proposed to overcome the limitations of multiple testing include false discovery rate (FDR), LD block-based corrections and permutation testing.

## Functional characterization

Functional characterization helps in discrimination of a causal SNP from an association due to linkage, and might serve to increment the present understanding of functional implication of the gene in disease mechanisms and drug response. Validation of SNPs that can alter the expression and activity of proteins and enzymes is of fundamental interest. Our current understanding of molecular biology dictates that most of the pathogenic SNPs reside in most conserved gene regions and location of the SNP, in particular gene regions, which defines its functional role. Typically, variants leading to a change in amino acid, i.e. nonsynonymous SNPs, lead to a change or loss in protein function, while the silent polymorphisms or synonymous SNPs exert their effect at the mRNA or protein expression level. Recently, the *ABCB1* 3435C>T (synonymous) genotypes were shown to alter conformations of P-glycoprotein, suggesting an effect on the protein folding.[24] Of late, keen interest has arisen in SNPs residing in the regulatory regions for the subtle changes caused by them in gene expression. For instance, Ufer *et al*. showed that the *ABCC2*-24T variant was significantly over-represented among nonresponding epilepsy patients. Although, the *ABCC2*-24C>T genotype did not affect hippocampal *ABCC2* expression, it was associated with increased *ABCB1* expression, suggesting its regulatory role.[25] Popular experimental approaches for *in vitro* functional characterization of SNPs include gene reporter assay, DNA footprinting, gel mobility shift assays, reverse transcriptase and quantitative polymerase chain reactions. Yet again, SNPs do not act in isolation, and point mutant constructs may hardly represent the various naturally occurring forms of the gene. Hence, functional studies for haplotypes are gaining prominence over single SNP characterization.

## Summary

The clinical findings presented in the current review strongly suggest the role of genetic variability on phenotypic manifestations of imbalances in the excitatory and inhibitory neurotransmission. Further, genetic polymorphisms from several candidate genes appear to modulate the risk factors for showing recurrent seizures, despite on adequate AED treatment.

However, due to limitations in the study designs ranging from epidemiological to statistical issues, we often end up with false results that might be difficult to replicate in populations with different ethnicities. Hence, there is an urgent necessitation of conducting large-scale genetic epidemiological studies with consistency in study designs by various research groups by taking into account all the confounding factors, including environmental and genetic variables. The advent of high-throughput genomic technologies coupled with strong bioinformatics and statistical tools would further enhance the chances of discovering genetic markers or their combinations with a high predictability for determining AED responsiveness and predisposition to side-effects in epilepsy patients.

If validated and replicated in populations from different ethnic backgrounds, these markers could aid in providing safe and efficacious treatment. Hence, such studies could further help in the development of individualized pharmacogenetic therapies with drug type, dose and duration tailored according to the genetic background of the patients. Further, using an interdisciplinary approach, including mRNA profiling and proteomics, such studies might be helpful for designing drugs targeting specific genes involved in AED disposition and action. In all, such a comprehensive integration of clinical evidence and methodological variability may re-invigorate reasons for optimism to the scientific community towards this naive field of “epilepsy pharmacogenomics.”
